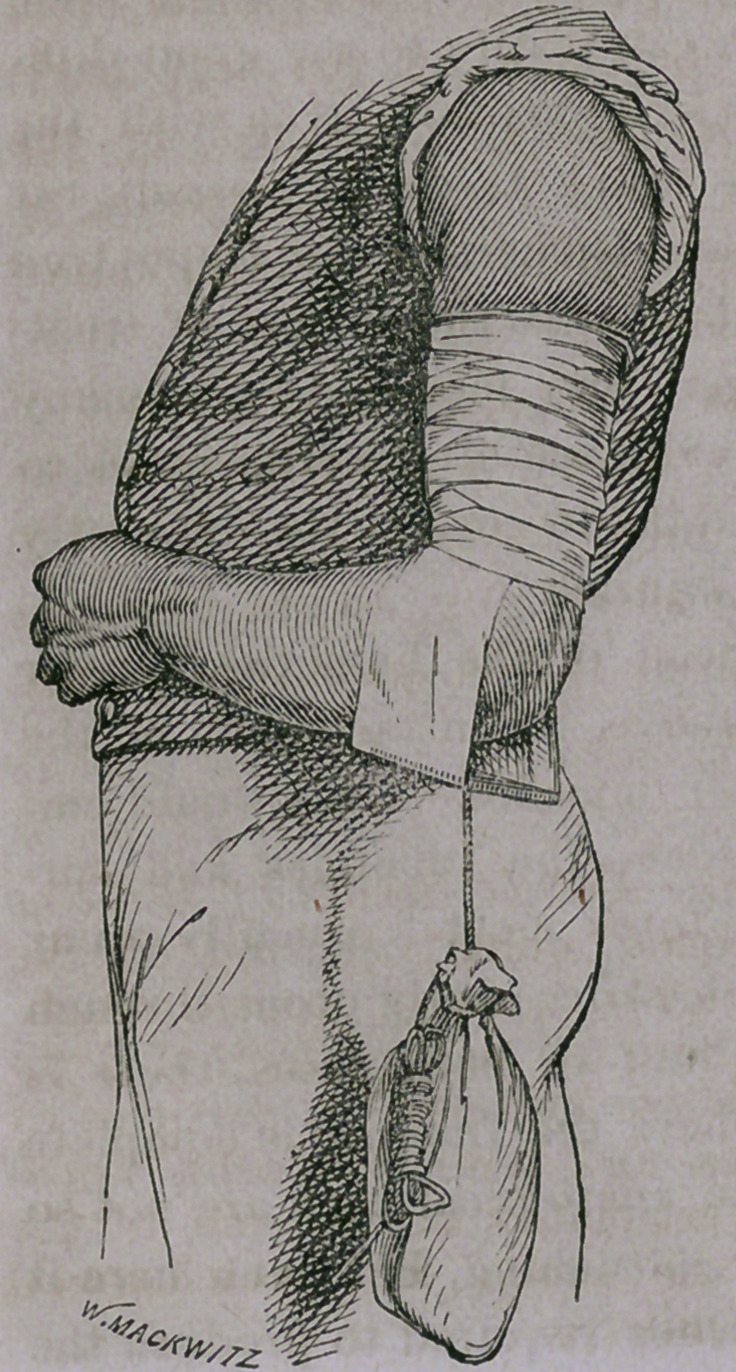# Method of Treating Fractures of the Olecranon Process, and Head of the Humerus

**Published:** 1868-08

**Authors:** E. A. Clark

**Affiliations:** Resident Physician, St. Louis City Hospital


### Miscellaneous.

### Method of Treating Fractures of the Olecranon Process, and Head of the Humerus.

By E. A. Clark, M. D., Resident Physician, St. Louis City Hospital.

### *Fractures of the Olecranon.*

I have found all the ordinary appliances in use for treating fractures of the olecranon so deficient in meeting the indications required, that I have been induced to devise the apparatus represented in the following woodcut, which is sufficiently simple to require but little description.

Fractures of the olecranon, as they usually occur towards the middle or base of the process, are generally attended with such a degree of displacement—especially in muscular subjects—that the ordinary method of applying narrow strips of cotton or cloth around the arm—both above and below the elbow—and approximating them by means of lateral strips, as recommended by Sir Astley Cooper and Amesbury, with the view of drawing down the upper fragment in apposition with the head of the ulna, and thus securing the condition most favorable for bony union, will necessarily require these bands to be so tight around the arm, at both points, as to arrest the circulation. This danger will be the more imminent in cases where is there much contusion and swelling of the soft parts, which, as might be expected, from the very nature of the violence or force required to produce this fracture, is almost always the case. The method of treatment recommended by these gentlemen is also objectionable in that they direct that the arm be kept in a straight position.

The apparatus above represented consists of a band of ordinary sole-leather about two inches in width, and of sufficient length to surround the arm, lined with cloth or chamois, and well padded with cotton or hair. In order to give the band additional firmness, and also to secure it around the arm, a strip of common harness-leather is stitched upon the outside, to the end of which two small buckles are attached, while the other end, which extends about three inches beyond the band, is split or cut into two straps to correspond with, and fasten into the buckles The band is fastened around the arm above the fractured process, and may be drawn to any degree of tightness necessary to bring the broken fragment down when traction is made upon it.

The same band may be used on either arm, and may be adapted to an arm of any size. On the outer side of this band, and one inch apart—one on each side of the olecranon—are two buckles or staples, which should be two inches in length, and three-fourths of an inch in width, and clinched on the inside of the leather band, from which they project at a right angle. These buckles or staples also have three bars across them, with two tongues made to turn either way.

In applying this apparatus the arm should be flexed at an angle of forty-five degrees, and a common pasteboard splint bent at that angle placed upon its anterior surface. The leather band is then buckled over this splint, just above the fragment of the olecranon, and the entire fore-arm is covered with a bandage to hold the anterior splint firm to the arm and thus prevent any movement of the elbow-joint, which, if allowed, would be constantly modifying the force exerted upon the fracture. A common buckskin glove is then placed upon the hand, to the anterior and posterior surfaces of which are attached two leather straps, which are to be buckled into the staples on the band. By buckling these straps over the bars at a greater or less distance from the band, and tightening them as required, we obtain the necessary amount of leverage to turn the lower edge of the band in upon the arm, and push the fractured process down before it.

By making traction upon these straps any degree of force may be exerted upon the band, necessary to draw the broken fragment down and hold it in perfect apposition with the head of the ulna.

It may be objected to this method of treatment, that the arm is held in a flexed position, thus increasing the space between the two fragments. But the advantage of this position is apparent for two reasons.

First, by flexing the arm to this extent the point of the olecranon is made more prominent, and, consequently, the band more surely adjusted, so as not to slip over it; while, again, the force exerted upon the band by the straps, directed at an angle of forty-five degrees from the axis of the humerus, renders the pressure still more secure above the point of the olecranon and prevents the possibility of its slipping back beneath the band.

The second reason for fixing the arm in this position is to relax the brachialis anticus muscle, the action of which, in cases where the fracture occurs low down, near the base of the olecranon, and especially in a muscular subject, when the arm is held in a perfectly straight position, evidently draws the head of the ulna forward, so that a portion of its fractured surface is in direct apposition with the articular surface of the lower end of the humerus; while if the detached fragment of the olecranon be forced down to its proper position it would not be in complete apposition with the upper end of the ulna, but would leave a triangular space in the articulation to be filled up by callous and thus produce more or less complete anchylosis of the joint.

This apparatus, when applied as described, is in no way painful to the patient, the band being padded in the inside and the pressure exerted by it on the anterior surface of the arm bearing upon the pasteboard splint; the only other pressure exercised is directly upon the olecranon, and that upon such a broad surface that sloughing need not occur in any case.

I have treated but one case with this apparatus, and with the following result:

A laboring man, aged 32 years, was admitted to hospital five days after receiving a fracture of the olecranon near its base. At the time of his admission he had an abscess as large as a hen's egg immediately over the point of the olecranon, resulting from a contusion received when the bone was fractured. The abscess was opened before the dressing was applied, and, notwithstanding all the pressure required to hold the bones in apposition was made upon the point over the abscess, it healed quite readily, and in seven weeks the apparatus was removed, leaving firm, bony union in the fracture, without the least deformity or displacement; and now—three weeks since—the patient has recovered almost perfect use of his arm.

No passive motion of the joint was allowed at any period of the treatment.

*Fractures of the Head of the Humerus.*

Every surgeon who has had much experience in treating fractures about the head of the humerus can testify to the great difficulty of maintaining the fragments in apposition, even with the most ingenious appliances, amongst which those of Desault, Sir A. Cooper, Fergusson, Erichsen, Welch, Richerard and Dupuytren are most generally used. The very fact that the means of treating these fractures have been changed and modified by so many distinguished surgeons, is sufficient evidence of the difficulties to be encountered in adapting any apparatus to correct the deformity most usually found to exist in these injuries.

In speaking of fractures of the head of the humerus, I refer only to that portion of the bone above the attachment of the latissimus dorsi and pectoralis major muscles. This would embrace—external to the capsular ligament—the tubercles and surgical neck, in the latter of which fractures most frequently occur from direct violence; yet fractures not unfrequently occur through the tubercles from the same cause, and in both cases, there is always more or less displacement, where the fracture is complete and not impacted. Fractures of the anatomical neck are not so often attended with displacement, or shortening, but even here it is not uncommon from the great violence required to produce the fracture, to find the capsular ligament ruptured and one or both fragments displaced. In all cases of fracture occurring outside of the capsule, where there is no impaction, there must be more or less displacement of the upper fragment from the contraction of the muscles attached about the tubercles. It is on this account that none of the appliances in ordinary use, such as pads in the axilla, and cap splints over the point of the shoulder, can be made effectual in maintaining the bones in apposition; because it is impossible to place any kind of compress in the axilla, that can be brought to bear upon the upper fragment, without producing an amount of pressure on the axillary vessels intolerable to the patient, while it would be a rare and peculiar fracture that could be kept in apposition, where the upper fragment and muscles attached to it, were allowed to go unrestrained, even though the shaft of the humerus might be maintained in its proper axis by the use of a pad in the axilla.

Where there is shortening of the limb, as is almost invariably the case in fractures at the surgical neck, none of these appliances could have the least influence in correcting such deformity, further than that the pressure from the bandages might control the contraction of the muscles.

In fracture of the anatomical neck with laceration of the capsular ligament attended with displacement, the pad in the axilla would be likely to increase the deformity, and it certainly could in no wise correct it.

The accompanying woodcut represents a method I have employed which is not open to the above objections. The appliance consists merely of two strips of adhesive plaster about three inches in width, applied to the internal and external surface of the arm as high as the upper part of the middle third of the humerus. These strips are bound to the arm by a roller bandage, and at their lower end, beneath the point of the elbow, are attached to a cord, to which a sand-bag is attached weighing, ordinarily, from three to four pounds.

The sand-bag, as represented in the diagram, is attached close to the point of the elbow when the patient wishes to walk about, by knotting the cord by which it is suspended, and when he lies in bed, the knot in the cord, as seen in the cut, is loosed, and the cord carried beneath the bed clothing over a small pulley placed at the foot of the bed, and in this way an equal extension is constantly kept up, whether the patient be confined to his bed or is able and prefers to walk about.

When using this apparatus for treating these fractures, I apply no other dressing, and entirely ignore the compress in the axilla, as useless, if not positively injurious. The constant traction upon the muscles soon exhausts their tonicity, so that they allow the bones to fall into their natural position, while the extension being constantly in the line of the axis of the humerus, it is quite impossible that any displacement should continue, either laterally or of an angular character, or that any shortening should result.

I have, as yet, treated but one case of fracture of the surgical neck of the humerus by this method.

The patient was a stout muscular man, aged 33 years, who had fallen some twelve feet, striking the point of the shoulder upon the ground, causing considerable contusion of the soft parts besides the fracture, which was considerably displaced, by the lower fragment projecting outward; there was also shortening to the extent of three-fourths of an inch. The patient complained of constant and severe pain at the point of fracture until the third day, when the above apparatus was applied, with the effect of relieving the pain almost instantly. At the end of seven weeks the dressing was removed and the union in the fracture found to be firm, without any displacement or shortening, and in ten days after, the patient was discharged from the hospital with perfect use of his arms.—*Humbolt Medical Archives.*

## Figures and Tables

**Figure f1:**
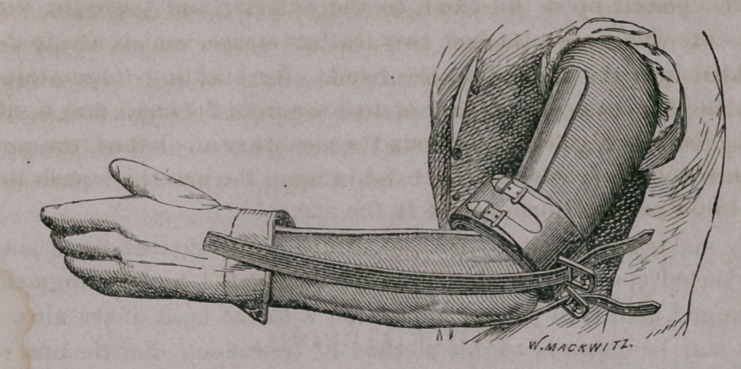


**Figure f2:**